# Different ankle muscle coordination patterns and co-activation during quiet stance between young adults and seniors do not change after a bout of high intensity training

**DOI:** 10.1186/s12877-015-0017-0

**Published:** 2015-03-04

**Authors:** Lars Donath, Eduard Kurz, Ralf Roth, Lukas Zahner, Oliver Faude

**Affiliations:** Department of Sport, Exercise and Health, University of Basel, Birsstrasse 320-B, 4052 Basel, Switzerland; Clinic for Trauma, Hand and Reconstructive Surgery, Division of Motor Research, Pathophysiology and Biomechanics, Jena University Hospital, Bachstrasse 18, 07743 Jena, Germany

**Keywords:** Upright stance, Postural control, Elderly, Exercise training, Balance, Risk of falling

## Abstract

**Background:**

Available evidence suggests that young adults and seniors use different strategies to adjust for increasing body sway during quiet standing. Altered antagonist muscle co-activation and different ankle muscle coordination patterns may account for this finding. Consequently, we aimed at addressing whether aging leads to changes in neuromuscular coordination patterns as well as co-activation during quiet stance. We additionally investigated whether a bout of high intensity interval training additionally alters these patterns.

**Methods:**

Twenty healthy seniors (age: 70 ± 4 y) and twenty young adults (age: 27 ± 3 y) were enrolled in the present study. In between the testing procedures, four consecutive high-intensity intervals of 4 min duration at a target exercise intensity of 90 to 95% HR_max_ were completed on a treadmill. The total center of pressure (COP) path length displacement served as standing balance performance outcome. In order to assess ankle muscle coordination patterns, amplitude ratios (AR) were calculated for each muscle (e.g. tibialis anterior (TA) [%] = (TA × 100)/(gastrocnemius medialis (GM) + soleus (SOL) + peroneus longus (PL) + TA). The co-activation was calculated for the SOL and TA muscles computing the co-activation index (CAI = 2 × TA/TA + SOL).

**Results:**

Seniors showed an inverted ankle muscle coordination pattern during single limb stance with eyes open (SLEO), compared to young adults (rest: GM, S: 15 ± 8% vs Y: 24 ± 9%; p = 0.03; SOL, S: 27 ± 14% vs Y: 37 ± 18%; p = 0.009; TA, S: 31 ± 13% vs Y: 13 ± 7%; p = 0.003). These patterns did not change after a high-intensity training session. A moderate correlation between amplitude ratios of the TA-contribution and postural sway was observed for seniors during SLEO (r = 0.61). Ankle co-activation was twofold elevated in seniors compared to young adults during SLEO (p < 0.001). These findings were also not affected by high intensity training.

**Conclusion:**

Increased ankle co-activation in the anterior-posterior plane and inverted ankle muscle coordination pattern merely occurred during single-leg stance. Seniors with decreased postural control showed higher TA contributions during SLEO. These neuromuscular changes are not affected by acute intermittent high intensity aerobic exercise.

## Background

Beside numerous external fall risk factors (e.g. lack of handrail, uneven terrain, twilight, obstacles), a body of evidence also emphasized that personal or intrinsic risk factors (e.g. visual impairment, medication intake, strength deficits of the lower limb, increased spatio-temporal gait variability and impaired balance performance) cause an elevated risk of falling in older people [1-3]. An aging-induced loss of vestibular, visual, somatosensory and neuromuscular function has been reported to result in deteriorated postural control with an increased postural sway during standing balance tasks in the elderly [4]. For example, diminished peripheral perception, delayed spinal reflex-loop recruitment, higher muscle activity levels with increased muscular co-activation and decreased spinal reflex transmission are considered to mainly account for deteriorated standing balance performance in seniors [5,6].

Postural sway serves as an appropriate outcome measure to examine postural control under static balance conditions. Thereby, single and double limb standing have been frequently applied [7]. The base of support and different sensory conditions can be modified in order to provide adequate progression [8]. Many studies indicated that the process of aging leads to declines in static postural control under various conditions [9].

Although muscle activity, postural control strategies (e.g. hip vs. ankle) and neuromuscular adaptations to balance training were previously examined in seniors [10], age-related differences of ankle muscle coordination and ankle-co-activation patterns have not yet been cross-sectionally examined during resting states and following an intense bout of aerobic exercise training. Several studies indicated that aerobic exercise training can lead to transient impairments of postural control [11-13]. It is, however, not clear to date whether underlying changes of muscle coordination account for these increases of postural sway. Although it seems well known that increased antagonist muscle co-activation in the elderly provides mechanical stability via stiffening joints and reducing degrees of freedom during balance tasks during several standing and walking tasks [10,14], these indices were not yet addressed after intense exercise. These altered age-related neuromuscular adaptations of ankle muscles to dynamic constraints are not necessarily present during static balance tasks or after an acute bout of intense exercise. Thus, the acute impact of intense exercise training on ankle muscle co-activation and ankle muscle coordination patterns during mono- and bipedal stance needs to be more clearly disentangled.

Against the aforementioned background, the present study aimed at investigating the relative contribution of ankle muscles to uni- and bipedal standing balance tasks in young and old adults during rest and after one bout of a single session of high-intensity interval training (HIIT). Due to previously reported age-specific postural strategies to maintain standing balance [15], we assume that seniors may also reveal different patterns of ankle muscle activity during rest compared to young adults. Since maximal exercise deteriorate postural control [11], we further presume that these ankle muscle coordination patterns in seniors are becoming more distinctive after exhaustive exercise and putative muscle-coordination pattern changes could be linked to acute exercise-induced elevations of postural sway.

We additionally intended to elucidate in which way intense exercise and aging affect ankle muscle co-activation. The present study may contribute to a better understanding of aging- and exercise-induced changes of ankle muscle coordination and co-activation. Thus, balance training approaches in seniors should be tailored to the specifities of aging-specific of ankle muscle coordination.

## Methods

### Study design and participants

Twenty healthy and physically active seniors older than 65 years (female/male: 12/8, age: 70 ± 4 y; body mass index: 25.0 (3.6) kg/m^2^ (mean (SD)); Body fat: 27.0 (9.1) %; physical activity: 10.9 (5.8) h/week) and twenty young and active adults (female/male: 9/11, age: 27 ± 3 y; body mass index: 22.4 (2.2) kg/m^2^; body fat: 17.9 (5.9) %; physical activity: 8.7 (4.1) h/week) were enrolled in the present study. All participants did not report any medication intake and health impairments that may affect balance and muscle activity testing. Seniors with diabetes, untreated hypertension (>180/110 mmHG), glaucoma, endoprosthesis, stroke, eczema and beta-blocker users were not included in the study. Static balance and muscle activity data were collected on two days one week apart. The first day served as familiarisation day for balance testing and to perform maximal exercise testing in order to derive maximum heart rate (HR_max_). The second testing day (one week later) was conducted to perform HIIT. Balance and muscle activity data were collected immediately before and after HIIT. The study was approved by the local ethics committee (Ethikkommission beider Basel (EKBB), approval number 257/12) and fulfilled the criteria of the declaration of Helsinki. All participants signed an informed written consent prior to the start of the study.

### Testings and analyses

Standing balance was tested during double limb stance with eyes closed (DLEC) and single limb stance with eyes open (SLEO) on a piezoelectric force-plate (Kistler, Type 9286, Winterthur, Switzerland). The task order was randomly assigned. Three attempts for each standing condition were achieved. The average of the performed trials was included into further analyses. Participants were standardizely instructed to perform without shoes, with feet placed shoulder width apart, hands attached at the hip and slightly bent knees. Additionally, they were controlled to keep the trunk in an upright position. Data were collected for 10 s at 40 Hz and analysed off-line using a low pass cut-off frequency of 10 Hz [7]. The total center of pressure (COP) path length displacement served as outcome measure.

Ankle muscle surface electromyography (mm. soleus, SOL; medial head of gastrocnemius, GM; tibialis anterior, TA; peroneus longus, PL) was measured according to the European recommendations for surface electromyography (SENIAM). To provide a skin conductance level of <5kΩ, the skin of the dominant leg, determined by means of the lateral preference inventory [16] was gently prepared (shavers and fine sandpaper). Raw electromyographic data were processed off-line using custom-made algorithms in MATLAB (The Mathworks, Natick, USA). After correcting possible offsets, removing 50 Hz and odd-numbered harmonics of the signals, a 20 Hz high-pass and a 400 Hz low-pass filter were applied, respectively (Kurz et al. [17]). Muscle activity was calculated with a moving root mean square (RMS) window of 0.2 s resulting in a total of 99 RMS values representing the envelope. Mean activity of the envelope was considered for further analysis, separately for each muscle, condition and trial. The average of the related EMG-signals of the three trials for each standing condition was included into analyses. In order to assess ankle muscle coordination patterns, the amplitude ratios (AR) were calculated [18] for each muscle (e.g. TA [%] = (TA × 100)/(GM + SOL + PL + TA)) [17]. The co-activation was calculated for the soleus (SOL) and tibialis anterior (TA) muscles computing the co-activation index (CAI = 2 × TA/TA + SOL). This equation assumes that TA is acting as an antagonist. Through this CAI a relative measure (arbitrary units, 0 indicating no co-activation) of TA contribution to total activation of both ankle muscles during the standing is provided.

### Acute intervention

Four consecutive high-intensity intervals of 4 min duration at a target exercise intensity of 90 to 95% HR_max_ were completed on a treadmill. Outcome variables were computed before and after the acute exercise intervention, The respective interval bouts were interspersed with active rest periods of 3 min at 70% HR_max_ [19]. In order to stay in between the required range of heart rate, treadmill inclination and velocity were adjusted if necessary. Seniors walked briskly with inclination to avoid locomotor or coordinative limitations. The young adults were allowed to run. Heart rate and gas exchange data were continuously measured.

### Statistics

Two separate 2 (age: seniors vs. young adults) × 2 (condition: pre vs. post) × 4 (muscle: GM, SOL, PL, TA) repeated measures analyses of variance (rANOVA) were computed for the COP as well as AR variables during SLEO and DLEC. Similarly, 2 (age: seniors vs. young adults) × 2 (condition: pre vs. post) rANOVA were calculated for the CAI during both stance conditions (SLEO, DLEC). To estimate practical relevance, partial eta squared (η_p_^2^) were calculated for the rANOVA of the respective interaction effects. Thereby, η_p_^2^ ≥ 0.01 indicates a small, ≥ 0.06 a medium and ≥ 0.14 a large effect [20]. In case of statistically significant interaction terms, Tukey HSD post hoc tests were calculated. Cohen’s d (standardized mean difference) was calculated for the between-group effect size for each muscle (trivial: d < 0.2, small: 0.2 ≤ d < 0.5, moderate: 0.5 ≤ d < 0.8, large: d ≥ 0.8). Correlations between total COP path lengths and activation ratios were examined using Pearson’s product–moment correlation. Data are reported as means with standard deviations. Gender, body mass index and physical activity were additionally considered as covariates and did not affected the results.

## Results

We did not find differences of COP path length displacements between groups from pre to post after conducting the acute HIIT intervention for DLEC (0.30 < p < 0.80, η_p_^2^ < 0.04) and SLEO (0.38 < p < 0.98, η_p_^2^ < 0.03).

**Figure 1:**
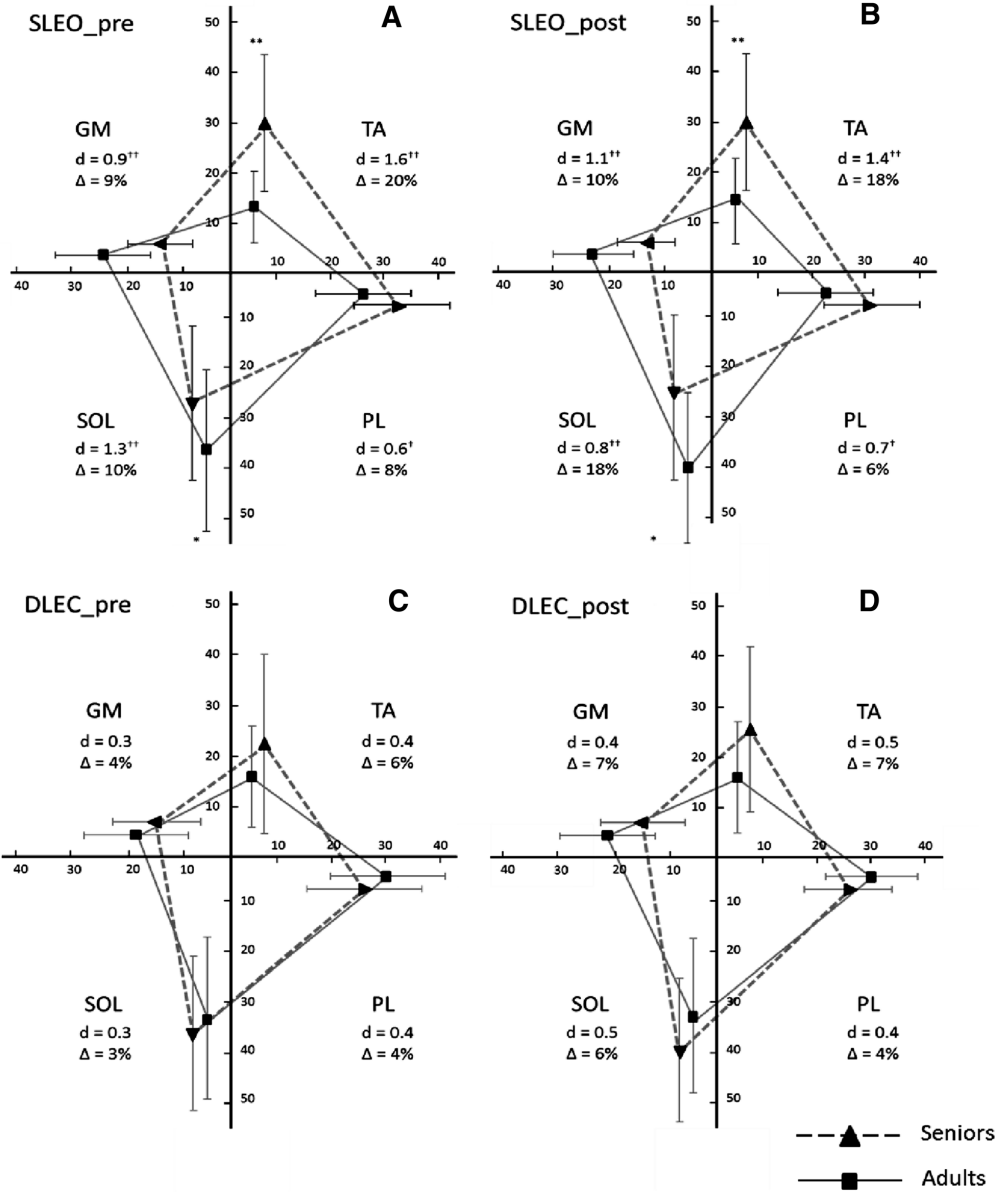
**Amplitude ratios for seniors (triangles with dotted lines) and young adults (squares with straight lines) for mm. tibialis (TA), peroneus longus (PL), soleus (SOL) and medial gastrocnemius (GM) as spider charts during single limb stance with eyes open (SLEO, top panels) and double limb stance with eyes closed (DLEC, bottom panels) indicated as percentage contribution with respect to the muscles measured here.** Cohen’d (0.2 ≤ d < 0.5, moderate†: 0.5 ≤ d < 0.8, large††: d > 0.8) and the mean group-difference for each muscle (delta: ∆) is provided. p < 0.05*, p < 0.01** and p < 0.001***.

No interaction effects including the factor “condition” (pre vs. post) were found for the amplitude ratios (AR) during SLEO (0.27 < p < 0.99; 0.001 < η_p_^2^ < 0.04). A large muscle × age interaction was observed (p < 0.001, η_p_^2^ = 0.20) for SLEO. Independent of HIIT, post hoc testing revealed higher amplitude ratios for TA in seniors (p = 0.001) and SOL (p = 0.048) in young adults (Figure 1A, B).

Regarding DLEC, neither interaction effects including the factor “condition” (0.41 < p < 0.49; 0.02 < η_p_^2^ < 0.03) nor muscle × age interactions were found (p = 0.14, η_p_^2^ = 0.05) (Figure 1C, D).

**Figure 2:**
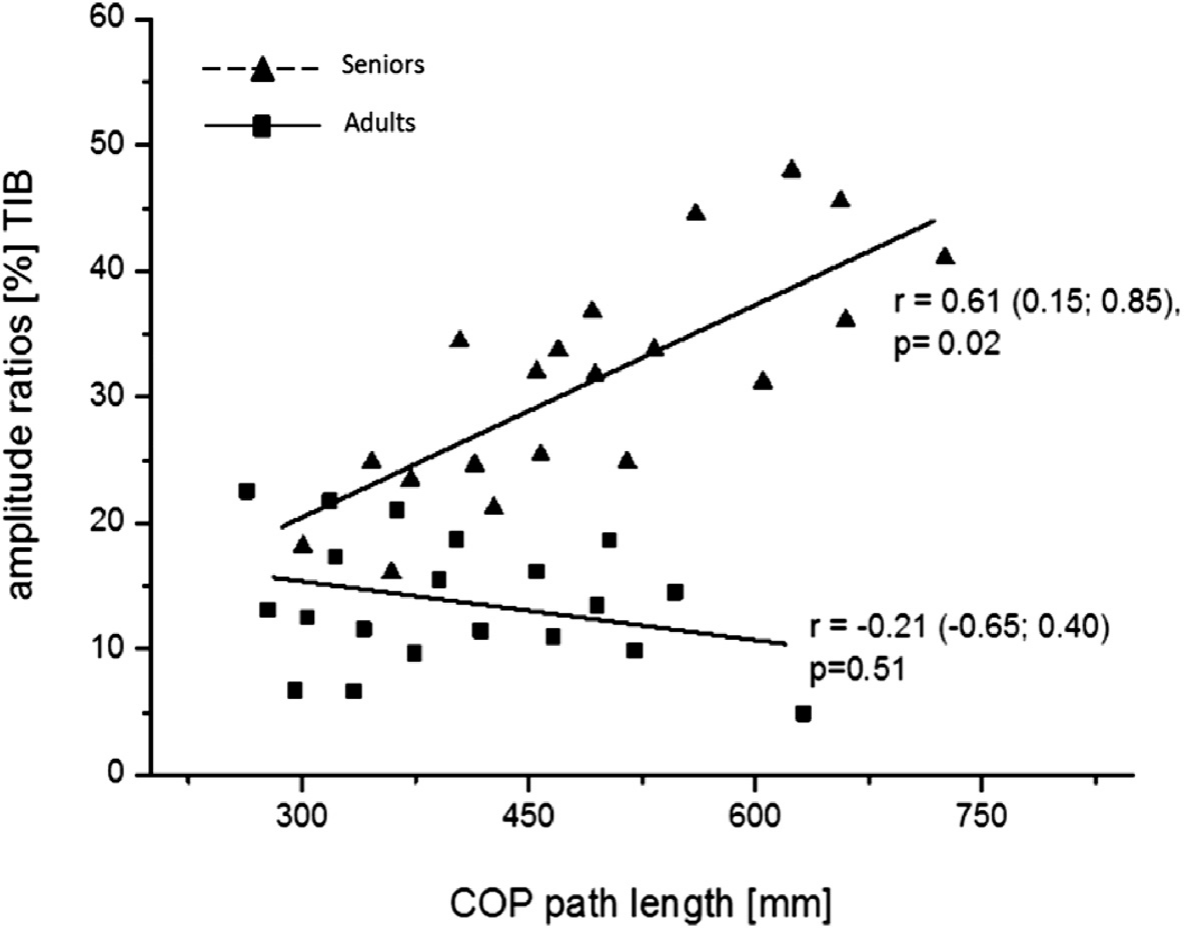
**Association between the amplitude ratios of the mm. tibialis anterior (TA) and postural sway for seniors (triangles) and young adults (squares) during single limb stance with eyes closed.** Correlation coefficient (r) is given with 95% confidence interval (CI).

A positive significant correlation between the amplitude ratios of TA and postural sway was observed in seniors but not in young adults (Figure 2) during SLEO. For the remaining muscles and for DLEC no such significant associations were found (−0.13 < r < 0.23; 0.34 < p < 0.78).

**Figure 3:**
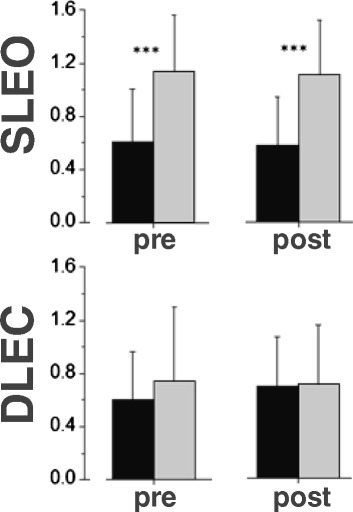
**Co-activation indices (CAI) of soleus and tibialis anterior muscles for adults (black bars) and seniors (grey bars) during single limb stance with eyes open before HIIT (A) and after HIIT (B) as well as during double limb stance with eyes closed before HIIT (C) and after HIIT (D) Data are indicated as means and standard deviations.** p < 0.05*, p < 0.01** and p < 0.001***.

Neither age × condition interactions (p = 0.69, η_p_^2^ = 0.005), nor age- (p = 0.57, η_p_^2^ = 0.01), nor condition-effects (p = 0.77, η_p_^2^ = 0.003) were observed for DLEC. Independent from the condition, a large age-effect (p < 0.001, η_p_^2^ = 0.44) was observed for SLEO (Figure 3).

## Discussion

The present study revealed for the first time that seniors showed an inverted ankle muscle coordination patterns during static single-limb standing compared to young adults. The occurrence and magnitude of the age-specific ankle muscle coordination patterns during SLEO and DLEC did not change after a single HIIT session in both groups. Whereas young adults seem to maintain single limb standing with a higher percentage contribution of posterior ankle muscles (soleus and gastrocnemius muscles), seniors ankle muscle coordination pattern rely on the anteriorly located tibialis muscle. Interestingly, COP path lengths did not changed after HIIT in both groups. Elevated postural sway during SLEO led to higher relative contribution of the tibialis muscle in the elderly. These patterns were, however, not present during double limb stance with closed eyes and did not alter after an intense bout of highly intense aerobic interval exercise. Similar findings were observed for the co-activation indices: Higher tibialis/soleus muscle co-activations during single limb standing balance which were not present during double limb standing and not affected by HIIT.

Regarding ankle muscle coordination patterns, amplitude ratios for the back-sided ankle muscles (GM + SOL) decreased for about 20% at rest and 30% after HIIT during SLEO in seniors compared to young adults. Merely considering percentage contribution does not clarify the origin of activity changes. Thus, tibialis AR increases might be a result of increased TA activity or, in turn, decreases of plantar flexor activity. In turn, the percentage contribution of the TA muscle increased for about 20% at rest and 18% after HIIT during SLEO. This inverted ankle muscle coordination pattern might reflect (a) the frequently used hip-strategy in seniors which is possibly accompanied with deteriorated postural control and/or (b) a higher TA/SOL muscle co-activation that has been reported to be associated with postural sway [21]. It seems plausible that a larger body sway modulated by the hip can provoke a backward shift of the lower extremity resulting in a greater activity of the TA. Similar shifts to a more TA-related coordination pattern have been observed in a clinical population with considerably deteriorated static balance performance during easy double-limb stance conditions with open eyes [17]. Corroboratively, a posterior shift of the COP has been proposed to account for this finding in the study of Kurz and coworkers [17]. However, we did not measure hip or ankle movements by kinematic analyses to address this issue with certainty. The positive linear relationship between postural sway and percentage TA contribution to SLEO stance underpins the importance of TA activation to adjust for increased body sway. One may indirectly conclude that decreased postural control with larger body sway cause higher TA/SOL co-activation and may induce the hip-strategy to maintain balance with a higher percentage contribution of the tibialis muscle. In turn, younger subjects might be more capable to adjust postural destabilizations by using the ankle strategy due to a potentially more efficient sensori-motor integration [22]. The hip-strategy, however, seems to allow a faster response to adjust COP displacements for the elderly. In contrast, independent from age, double leg standing with suppressed visual feedback on a stable base of support does not change ankle muscle activation patterns. It seems likely that the difficulty of tasks leading to increased sway path may result in changes in muscle activity patterns due to changes in the adjustment strategy (ankle vs. hip).

Taking the results after the acute HIIT intervention into account, the inverted ankle muscle coordination pattern did not change as a result of exhaustive endurance exercise, neither in young nor in healthy elderly persons. Although several studies revealed increased postural sway after moderate cycling [13] and exhaustive walking [11] in seniors, an indirect link between exercise-induced increases of postural sway and changes of ankle muscle coordination patterns should be handled with caution. It seems more likely that age-induced changes of postural strategies account for changes of relative muscle activity contribution of the ankle, independent from the mode of exercise. Thus, it would be reasonable to assume that muscle fatigue after endurance exercise lead to depressed proprioception (decreased spindle afferent fibre discharge) with decreased γ-motoneurone activity consistently in all muscles that are involved during upright stance [23,24]. To date, however, no studies investigated the influence of acute and chronic exercise on muscle coordination pattern. These studies are mandatorily required to better understand the interrelation between aging, balance training and postural control strategies and muscle coordination. Therefore, dynamic, kinematic and electromyographic methods should be integratively employed in future studies.

Increasing co-activation has been frequently reported in the elderly [25]. Elevated co-activation is considered to increase joint stiffness and a stiffer joint, in turn, shows reduced degrees of freedom that need to be integratively organized by the sensori-motor system [14]. As a consequence, higher joint stability during dynamic tasks has been assumed. There is no conclusive evidence available on the interrelation between co-activation, acute exercise effects and postural sway under static balance condition [10,26,27]. It is still unclear whether exercise affects co-activation and elevated co-activation leads to higher postural sway or, in turn, decreased postural control lead to higher co-activation, also after exercise. Although Collins et al. as well as Laughton and coworkers emphasized early, that increased muscle co-activation may account for increases of short-term postural sway [10,28], only few randomized controlled trials examined the interdependency between improvements of balance performance and changes of ankle co-activation in the elderly [26]. Interestingly, the latter study revealed that training-induced balance improvements lead to decreased co-activation under dynamic balance conditions (e.g. functional reach). It appears likely that elevated co-activation during balance tasks are more a compensatory strategy to counteract aging-related muscle weakness or detraining [10]. This seems to be particularly true for the tibialis anterior muscle. The neuromuscular properties (e.g. firing rate twitch contraction duration) of the TA muscle are tremendously changing with aging and are additionally regarded as major contributors to fall incidences [29,30]. Although ageing is accompanied with muscle atrophy, force generation of plantar flexors seems to be unaffected [31]. However, due to basically lower muscle volume of TA compared with triceps surae muscle group [32] even a high CAI does not necessarily reflect a balanced mechanical state [33]. Increased co-activation of the TA and SOL has been reported to facilitate muscle spindle proprioceptive function by increasing fibre recruitment and firing rates of primary afferents [34]. Since muscle fatigue seems to diminish muscle spindle function, we expected higher co-activation after exercise [23]. However, an acute bout of intense exercise does not seem to adversely affect proprioceptive function. Thus, increases of postural sway after intense exercise does not necessarily affect the co-activation interplay between the tibialis and soleus muscles.

However, some limitations need to be addressed. The included subjects were highly active and did not reflect a very fall-prone population. The generalizability and transferability to older and frail subjects might be limited. It is reasonable to assume that frail and less active older adults might show inverted activity patterns during double limb stance. This finding is particularly important since muscle activity data provide large baseline variability. Small effects cannot be detected sufficiently. Although shorter data collection periods (<20 seconds) can yield a transient time signal shift that may affect COP assessment [35], we refrained from collecting longer time frames since a majority of seniors are unable to stand longer than 10 seconds [36]. However, the acute effects during SLEO were large and not affected by this statistical power issue. Moreover, kinematic measures were not included. Also EMG applications of the trunk muscles were not employed. Thus, we can only speculate on potential underlying postural strategies. Further cross-sectional and longitudinal studies need to be conducted in order to address changes of trunk muscle activity and kinematics in more frail subjects before and after balance training interventions.

## Conclusion

Ankle joint muscle coordination and TA/SOL co-activation during single leg standing with open eyes seems to be affected by age but not inevitably by intense aerobic exercise. Although several studies revealed increases of postural sway after submaximal cycling or exhaustive walking in seniors, an increased exercise-induced body sway is neither linked to modulated ankle muscle coordination pattern nor co-activation. Although this finding might be of relevance for balance training programs in seniors, training recommendations from young adults are not necessarily applicable to seniors and vice versa. It might be hypothesized that a progressive training approach for seniors should start with different forms of supported single leg stance with open eyes in order to overcome the altered activity of ankle muscles and ensure a more stable situation for advanced postural tasks. Adequately developed future balance training regimes could then improve postural strategies in the elderly to better adapt to external perturbations and balance study implications for balance training to improve fall-risk factors in seniors.
